# Proton pump inhibitors and myocardial infarction: an application of active comparators in a self-controlled case series

**DOI:** 10.1093/ije/dyac196

**Published:** 2022-10-19

**Authors:** Celine S L Chui, Ka Shing Cheung, Jeremy P Brown, Ian J Douglas, Ian C K Wong, Esther W Chan, Angel Y S Wong

**Affiliations:** School of Nursing, University of Hong Kong, Hong Kong SAR, China; School of Public Health, University of Hong Kong, Hong Kong SAR, China; Laboratory of Data Discovery for Health (D24H), Hong Kong Science and Technology Park, Sha Tin, Hong Kong SAR, China; Department of Medicine, University of Hong Kong-Shenzhen Hospital, Hong Kong SAR, China; Department of Medicine, Li Ka Shing Faculty of Medicine, University of Hong Kong, Hong Kong SAR, China; Faculty of Epidemiology and Population Health, London School of Hygiene and Tropical Medicine, London, UK; Faculty of Epidemiology and Population Health, London School of Hygiene and Tropical Medicine, London, UK; Laboratory of Data Discovery for Health (D24H), Hong Kong Science and Technology Park, Sha Tin, Hong Kong SAR, China; Centre for Safe Medication Practice and Research, Department of Pharmacology and Pharmacy, Li Ka Shing Faculty of Medicine, University of Hong Kong, Hong Kong SAR, China; Research Department of Practice and Policy, UCL School of Pharmacy, University College London, London, UK; Laboratory of Data Discovery for Health (D24H), Hong Kong Science and Technology Park, Sha Tin, Hong Kong SAR, China; Centre for Safe Medication Practice and Research, Department of Pharmacology and Pharmacy, Li Ka Shing Faculty of Medicine, University of Hong Kong, Hong Kong SAR, China; Faculty of Epidemiology and Population Health, London School of Hygiene and Tropical Medicine, London, UK

**Keywords:** Proton pump inhibitors, self-controlled case series, active comparator, H2 receptor antagonist

## Abstract

**Background:**

Previous studies investigating potential cardiovascular adverse events of acid-suppressing drugs are susceptible to protopathic bias and confounding. We aimed to investigate the association between short-term risk of myocardial infarction (MI) and proton pump inhibitors (PPIs) using a self-controlled case series (SCCS) with an active comparator.

**Methods:**

We conducted a SCCS using a population-wide database from Hong Kong from 2003–2014. Adult with ≥1 outpatient oral PPI prescription or H_2_ receptor antagonist (H_2_RA) and MI during the observation period were included. We used both simple ratio and effect modifier approaches to SCCS with active comparators to obtain comparator adjusted estimates.

**Results:**

A total of 2802 and 1889 people with MI who had exposure to PPIs and H_2_RA were included respectively. We observed a higher risk of MI during days 1–14 following the start of PPI prescription (Incidence rate ratio (IRR): 2.30, 95% confidence interval (CI): 1.76–3.00) versus baseline. Similarly, we observed a higher risk of MI during days 1–14 following the start of H_2_RA prescription (IRR: 2.46, 95%CI: 1.92–3.16) versus baseline. In the novel SCCS analyses, comparator adjusted estimates were 0.93 (95%CI: 0.57–1.30) and 0.83 (95%CI: 0.58–1.20) during days 1–14 in simple ratio and effect modifier approach, respectively.

**Conclusions:**

We observed no difference in risk of MI associated with PPIs compared with baseline using H_2_RA as the active comparator. The elevated risk of MI associated with PPIs is likely due to protopathic bias. More studies are required to explore the feasibility of using active comparators in SCCS to address protopathic bias in addition to confounding.

Key Messages

- We identified 405 137 patients prescribed outpatient proton pump inhibitor (PPI) prescriptions and 2 002 000 patients prescribed H_2_ receptor antagonist (H_2_RA) prescriptions from 2003 to 2014 in Hong Kong using population-based datasets from the Clinical Data Analysis and Reporting System.
- In conventional self-controlled case series (SCCS), we observed a higher risk of myocardial infarction (MI) associated with PPIs within 14 days following the start of prescription. Similar temporal pattern was also observed for H_2_RA.
- In novel SCCS with an active comparator, there was no difference in risk of MI associated with PPIs compared with baseline using H_2_RA as the active comparator. The elevated risk of MI associated with PPIs is likely due to protopathic bias.
- This study does not support the evidence of a short-term elevated risk of MI associated with PPIs.
- Active comparators in SCCS may be an option to address protopathic bias in addition to confounding.

## Introduction

Proton pump inhibitors (PPIs) are one of the most prescribed medicines worldwide for the treatment of acid-related disorders, such as gastroesophageal reflux disease, dyspepsia and peptic ulcer disease.^1^ Despite their well-tolerated safety profile, some studies showed an increased cardiovascular risk associated with PPIs.^2^ An in-vivo study and a small clinical study suggested that PPIs may inhibit an enzyme (dimethylarginine dimethylaminohydrolase) to metabolize asymmetrical dimethylarginine, which could lead to adverse vascular effects.^3^^,^^4^

However, a recent large-scale randomized controlled trial reported no difference in risk of cardiovascular events in long-term PPI users compared with placebo over 3 years of follow-up.^5^ Although it demonstrated a long-term cardiovascular safety of PPIs with strong evidence, it did not report a potential time-varying magnitude effect,^6^ specifically the period shortly after taking PPIs. Therefore, the short-term cardiovascular risk following PPI therapy remains uncertain.

Some observational studies investigated the cardiovascular risk in different follow-up periods and reported contradictory findings on the cardiovascular effects of PPIs.^7–17^ Among them, some studies repeated the analyses using the alternative treatment H_2_ receptor antagonists (H_2_RAs) as a negative exposure control which should have shown no association with myocardial infarction (MI), but harmful associations were found.^7^^,^^10^^,^^13^^,^^17^ As cardiovascular conditions including MI may present with gastrointestinal-like manifestations, PPIs and H_2_RAs may be prescribed for patients who first presented with MI. This might lead to reverse causality (or protopathic bias) when such association is analysed using electronic health records where MI would be undiagnosed at first and then confirmed and recorded after patients were prescribed PPIs or H_2_RAs.

To account for health differences between people, the self-controlled case series (SCCS) method compares the rate of outcomes in different periods within the same individual.^18^ Recently, a novel SCCS method for active comparators has been further developed to reduce the time-varying confounding by indication, where the effect estimate for the active comparator, with no hypothesized causal association with the outcome, is incorporated into the analysis to quantify the association between the drug of interest and outcome relative to an active comparator.^19^ While PPIs and H_2_RAs as an active comparator are both susceptible to protopathic bias in this case scenario, we therefore proposed that this novel SCCS method could mitigate the effect of potential protopathic bias, in addition to minimizing confounding.

We aimed to investigate the association between PPIs and MI using the novel SCCS with an active comparator (H_2_RAs) in this population-based study.

## Methods

### Study design

We applied both a conventional SCCS design and an SCCS using an active comparator in this study.

### Data source

We used data from the Clinical Data Analysis and Reporting System which is managed by the Hospital Authority in Hong Kong. The Hospital Authority provides public health care services to more than 7 million Hong Kong residents. The database contains patients’ demographic characteristics and clinical records of diagnoses, operations, prescriptions and visits to accident and emergency departments, hospitals and outpatient clinics. Anonymized patient identifiers were generated to link all medical information including diagnostic and prescribing data. This database has been used to conduct drug safety studies associated with gastroenterological, cardiovascular, antimicrobial and psychotropic medications and COVID vaccines.^20–24^

### Participants

The SCCS includes people who have an outcome of interest during the observation period. It can control for time-invariant confounding by comparing the incidence rates during pre-specified risk periods with baseline within an individual.^18^ We therefore identified people aged ≥18 years at cohort entry with both oral outpatient PPI prescriptions and first recorded MI event as the principal diagnosis in either an inpatient setting (date of hospital admission) or a visit to an accident and emergency department during the study period (1 January 2003 to 31 December 2014). We excluded people who had a history of MI before the study period. We identified MI events using International Classification of Diseases, Ninth Revision (ICD-9) code 410. The positive predictive value for MI was previously shown to be high (85.4%; 95% CI: 78.8%–90.6%) in the Clinical Data Analysis and Reporting System.^25^

To identify incident oral outpatient PPI users, we excluded patients who had any PPI therapy 3 years before the study start date. Furthermore, we excluded patients who had inpatient or non-oral PPI therapy before the first oral outpatient PPI therapy.

### Conventional self-controlled case series

In the conventional SCCS, we compared the incidence rate of first MI during risk periods with the baseline within an observation period. The observation period started from 1 year after the patient entered the database (cohort entry) and ended at the earliest of the study end date, death or receiving any non-oral or inpatient PPI therapy after the first oral outpatient PPI therapy. To investigate the short-term cardiovascular risk of PPIs, we defined risk periods as follows: Days 1–14, Days 15–30 and Days 31–60 since the prescription start date (prescription date). To correct the estimates if the exposure is event dependent, we included a 14-day pre-exposure period before each prescription. All other periods were defined as baseline (Supplementary Figure S1, available as Supplementary data at *IJE* online). Any remaining exposed time longer than the predefined risk periods (i.e. exposed time to PPIs from Days 61+ if a PPI prescription was longer than 60 days) were removed from baseline.

We repeated the conventional SCCS analysis using H_2_RAs as exposure because H_2_RAs share similar indications to PPIs. Based on the current evidence, no plausible biological mechanism suggests that H_2_RAs may lead to MI. We searched PubMed on 5 May 2022 using keywords and Boolean operators: (histamine 2 receptor antagonists or cimetidine or famotidine or nizatidine or ranitidine) and (myocardial infarction or heart attack), yielding 76 studies. Of them, only two studies were identified to be relevant to the potential mechanism, if any. Results showed that H_2_ receptor activation could exacerbate myocardial injury.^1^ One study demonstrated a protective effect of histamine against myocardial injury for an H_2_RA.^2^ However, no clinical studies have been found to support such association. Therefore, any positive association found might imply that it could be attributed to symptoms that prompted the use of antacids and which were also risk factors for MI. We randomly selected a subset of patients who had H_2_RAs during the study period, to match the same number of identified PPI users. The inclusion and exclusion criteria and definitions of risk periods and observation period were the same as those in the analysis for PPIs. A patient could be included in both PPI and H_2_RA cohorts (3.3%).

We estimated incidence rate ratios (IRR) with age adjustment by dividing all periods into 5-year age bands, using conditional Poisson regression. Where there were treatment breaks of ≤30 days, patients were assumed to be exposed to PPIs/H_2_RAs continuously, accounting for any potential medication stockpiling and non-adherence.

### Novel SCCS using an active comparator

This study method has been used to study the association between penicillin and venous thromboembolism, using roxithromycin as comparator, and has been shown to mitigate time-varying confounding by indication.^19^ In our study, using the IRRs for PPIs and H_2_RAs estimated separately in the conventional SCCS, we further estimated the comparator-adjusted IRRs with the novel SCCS method using both the simple ratio approach and effect modifier approach.

Using the simple ratio approach, we calculated the comparator-adjusted IRRs by dividing (IRRs for PPIs) by (IRRs for H_2_RAs) for each risk period, followed by computing the 95% confidence interval (CI) using the Wald test-based method (see Supplementary Method S1, available as Supplementary data at *IJE* online).^19^ With the effect modifier approach, we included in the model terms for either drug exposure period and being a PPI user, and an interaction term between either drug exposure period and being a PPI user^8^ (see Supplementary Method S2, available as Supplementary data at *IJE* online). We then obtained the comparator-adjusted IRRs from the interaction term with its own 95% CI.

### Sensitivity analysis

In response to the peer reviewers’ comments, we further conducted sensitivity analyses to test the robustness of the results. First, we repeated the analyses with a finer age adjustment, by dividing all periods into 2-year age bands. This could reduce the impact of time-varying confounding. Second, we removed overlapped cohorts of PPI and H_2_RA users as a sensitivity analysis. Third, we removed PPI users who were prescribed an outpatient H_2_RA 60 days before or after their start of PPI prescription and H_2_RA users who were prescribed an outpatient PPI 60 days before or after their start of H_2_RA prescription, to test any potential effect of drug switching. We also removed PPI users who were prescribed an outpatient H_2_RA during any risk periods and H_2_RA users who were prescribed an outpatient PPI during any risk periods, as another sensitivity analysis.

Data management was performed using SAS software, version 9.4 (SAS Institute), with analysis carried out using Stata/MP 16.1.

## Results

### Proton pump inhibitors

We identified 405 137 patients who received 2 341 849 outpatient PPI prescriptions during the study period. Supplementary Table S1 (available as Supplementary data at *IJE* online) shows the proportion for each type of PPI.

**Figure 1:**
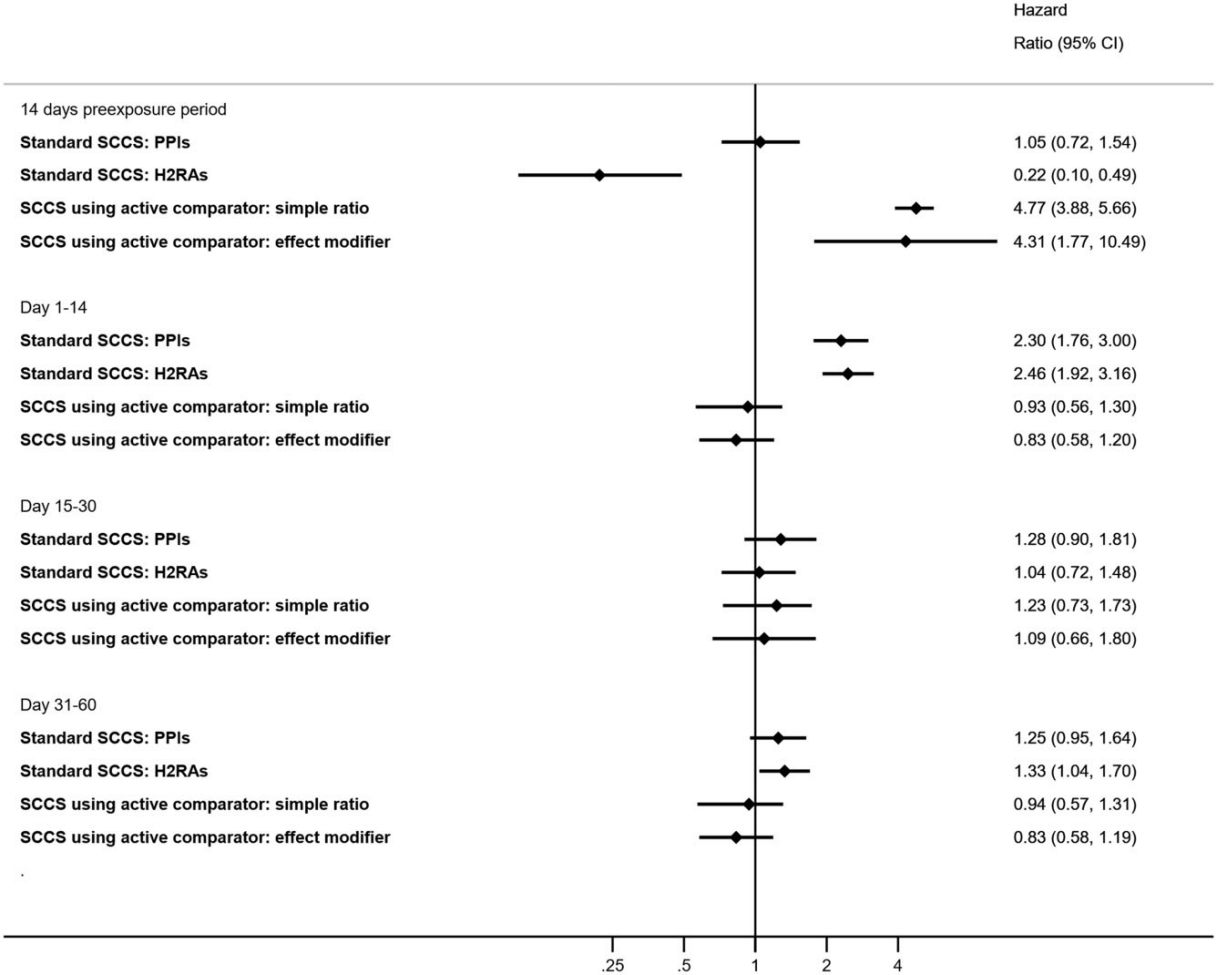
Summary of the results in the conventional and novel self-controlled case series analyses. CI, confidence interval; SCCS, self-controlled case series; PPI, proton pump inhibitor; H_2_RAs, H_2_ receptor antagonists

A total of 2802 patients who had both an outpatient PPI prescription and incident MI during the observation period were included. Their mean age (SD) at cohort entry was 62.3 (13.6) years. Men accounted for 64% of the study cohort. Supplementary Figure S2 (available as Supplementary data at *IJE* online) shows the flow of subject inclusion. Supplementary Table S2 (available as Supplementary data at *IJE* online) shows the health characteristics of PPI users on the prescription start date. Supplementary Table S3 (available as Supplementary data at *IJE* online) shows patterns of days of supply for prescription, observation period, risk periods and baseline periods among PPI users. We observed a higher risk of MI associated with PPIs during Days 1–14 (IRR: 2.30, 95% CI: 1.76–3.00) (Figure 1 and Table 1) but it did not persist during Days 15–30 (IRR: 1.28, 95% CI: 0.90–1.81) or during Days 31–60 (IRR: 1.25, 95% CI: 0.95–1.64) vs baseline. No increased risk was found in the pre-exposure period vs baseline (IRR: 1.05, 95% CI: 0.72–1.54).

**Table 1:** Incidence rate ratios for myocardial infarction associated with proton pump inhibitors and H_2_ receptor antagonists, respectively

	No. of events	Total person-years	Incidence rate ratio (95% CI)
**Proton pump inhibitors (*N *= 2802)**			
Baseline	2631	23 829	Ref
14 days pre-exposure period	27	127	1.05 (0.72–1.54)
Days 1–14	56	123	2.30 (1.76–3.00)
Days 15–30	33	133	1.28 (0.90–1.81)
Days 31-60	55	234	1.25 (0.95–1.64)
**H_2_ receptor antagonist (*N *= 1889)**			
Baseline	1715	14 950	Ref
14 days pre-exposure period	6	140	0.22 (0.10–0.49)
Days 1–14	67	139	2.46 (1.92–3.16)
Days 15–30	31	155	1.04 (0.72–1.48)
Days 31–60	70	274	1.33 (1.04–1.70)

### H_2_ receptor antagonists

We identified 2 002 000 patients who received outpatient 13 197 067 H_2_RA prescriptions during the study period. Supplementary Table S4 (available as Supplementary data at *IJE* online) shows the proportion for each type of H_2_RA.

Among a random subset (*N *= 405 137), we included 1889 patients who had both exposure to H_2_RA and MI within the observation period. Their mean age (SD) at cohort entry was 61.2 (13.8) years. Men accounted for 69% of the study cohort. Supplementary Table S2 shows the health characteristics of H_2_RA users on the prescription start date. In general, they were less likely to have comorbidities and cardiovascular drugs issued in the past 6 months, compared with PPI users. Supplementary Table S4 shows patterns of days of supply for prescription, observation period, risk periods and baseline periods among H_2_RA users, which were similar to those of PPI users. Similarly, we observed a higher risk of MI associated with H_2_RA during Days 1–14 (IRR: 2.46, 95% CI: 1.92–3.16) (Figure 1 and Table 1), but it did not persist during Days 15–30 (IRR: 1.04, 95% CI: 0.72–1.48). A marginal higher risk of MI associated with H_2_RAs was observed during Days 31–60 (IRR: 1.33, 95% CI: 1.04–1.70) vs baseline. A lower risk of MI associated with H_2_RAs was found in the pre-exposure period vs baseline (IRR: 0.22, 95% CI: 0.10–0.49).

### Novel active comparator analyses

Using the simple ratio method, we observed no difference in comparator-adjusted estimates for PPIs (IRR: 0.93, 95% CI: 0.57–1.30) during Days 1–14 vs baseline (Figure 1 and Table 2). Using the effect modifier approach, we also observed no difference in risk of MI associated with PPIs (IRR: 0.83, 95% CI: 0.58–1.20) during Days 1–14 vs baseline. Similar to the conventional SCCS, we observed no difference in risk of MI associated with PPIs in all other pre-specified risk periods compared with baseline.

**Table 2.** Novel self-controlled case series using active comparators.

	Simple Ratio Estimate		Effect Modifier Estimate	
	Estimate	95%CI	Estimate	95%CI
14 days pre-exposure period	4.77	1.96–11.62	4.31	1.77–10.49
Days 1-14	0.93	0.65–1.35	0.83	0.58–1.20
Days 15-30	1.23	0.75–2.03	1.09	0.66–1.80
Days 31-60	0.94	0.65–1.35	0.83	0.58–1.19

### Sensitivity analysis

Results of all sensitivity analyses were similar to those in the main analyses (Supplementary Tables S5–S12, available as Supplementary data at *IJE* online).

## Discussion

### Summary

Based on routinely population-based collected data, our study showed a higher incidence of MI in the first 14-day exposure to PPIs compared with baseline in the conventional SCCS. A similar temporal pattern was also found for H_2_RAs, demonstrating the possibility of protopathic bias. However, using the novel SCCS method with an active comparator, our study showed that there was no difference in risk of MI during the first 14 days associated with PPIs, as both comparator-adjusted estimates based on either simple ratio approach or effect modifier approach were close to null.

### Findings in context

In line with our study, some studies showed no difference in the risk of cardiac events associated with PPIs compared with H_2_RAs using a cohort study design with a propensity score method^14^^,^^15^ or case-crossover design.^11^^,^^12^ In contrast, some cohort studies suggested a cardiovascular risk of PPIs in both the short and the long term.^8–10^^,^^16^ Contrary to our result, long-term increased risk of MI and cardiovascular mortality (2–5 years) was also found.^8^ Given that a recent randomized controlled trial provided strong evidence to support long-term cardiovascular safety of PPIs,^5^ the unmeasured confounding in these between-individual comparison studies, resulting from differences between PPI and non-PPI users, might explain an apparently harmful association. This highlights the importance of the use of self-controlled methods to reduce important health differences between individuals.

However, observational studies including a conventional self-controlled method can still be susceptible to protopathic bias. In this study, we found a higher incidence of MI in the first 14-day exposure to PPIs compared with baseline, with similar patterns observed for H_2_RAs. As H_2_RAs were a negative control exposure, the positive findings strongly support that the association between PPIs and MI was likely to be non-causal. In our literature search, some in-vivo studies suggested H_2_ receptor activation could exacerbate myocardial injury and there could be a protective effect of H_2_RAs against myocardial injury.^26^^,^^27^ If it is confirmed in a clinical setting, this might further support our hypothesis that a higher risk of myocardial infarction observed following acid-suppressing drugs could be explained by the initial symptoms that prompted the use of acid-supressing drugs. We are not aware of pharmacological actions of acid suppression which could lead to MI. Other observational studies evaluated the risk of MI among H_2_RA users for comparison and found harmful associations. Two nested case-control studies showed a higher risk of MI in both PPI and H_2_RA users in a 90-day exposure window^13^ and in a 5-day exposure window, respectively.^17^ Similar to our study, a self-controlled risk interval study reported an increased risk of MI during a 4-week prescription vs another 4-week control period for both PPIs [odds ratio (OR): 1.5, 95% CI: 1.4–1.7] and H_2_RAs (OR: 1.8, 95% CI: 1.7–1.9).^7^ These studies suggest that the link between acid-suppressing drugs (i.e. PPIs or H_2_RAs) and MI was likely due to protopathic bias: as most of the PPI and H_2_RA prescriptions were given empirically to relieve gastrointestinal symptoms, the initial symptoms which prompted the use of acid-suppressing drugs might be due to MI, which was diagnosed at a later stage after the further clinical work-up examination. Therefore, it is necessary to consider protopathic bias in self-controlled study designs, and the novel SCCS with an active comparator can be used to mitigate protopathic bias.

Notably, we observed a lower risk of MI associated with H_2_RAs during the pre-exposure risk window in our analysis. As one of the assumptions of the SCCS design is that the occurrence of an event does not alter the probability of subsequent exposure, removing a pre-exposure risk period from baseline could correct the distortion of baseline incidence arising from short-term dependency. A lower risk of MI associated with H_2_RAs in the pre-exposure period might imply that clinicians were unlikely to prescribe outpatient H_2_RA prescription to people with a recent MI event. More importantly, it demonstrates the importance of removing the pre-exposure period from baseline to avoid the depletion of baseline rate.

### Strengths and limitations

To our knowledge, this is the first study to demonstrate that the novel SCCS method with an active comparator can be used to address protopathic bias if the comparator drug is also subject to a similar extent of protopathic bias. We also further adjusted for age as a time-varying confounder in 5-year and 2-year age bands. This study used routine clinical data for greater generalizability. The use of a negative control exposure provided robust internal validation. Moreover, this database was shown to have a high positive predictive value for MI,^25^ which ensures the accuracy of the outcome ascertainment.

We recognize possible limitations. First, we used prescription data to identify drug exposure, so it is unknown whether the patients took the drugs and thus may lead to misclassification bias. To reduce such bias, we assumed continuous exposure for treatment breaks of ≤30 days. In addition, the data from the private health care sector is not available in the Clinical Data Analysis and Reporting System. Further, the supply of H_2_RAs over the counter is not captured. If the person-time was misclassified as the baseline period due to over-the-counter H_2_RAs, the estimate would only remain unchanged, assuming that H_2_RAs have no effect on MI. It is also possible that the misclassification bias might inflate the estimate if H_2_RAs have a potential protective effect against MI. However, we included patients who visited the public health care sector at least twice during the observation period. Given the low consultation fee and drug cost, the included patients were likely to use public health care services rather than private health care services. Further, depending on the prescribing patterns in Hong Kong, most of the PPI and H_2_RA prescriptions we included were pantoprazole and famotidine, respectively. Further research is required to investigate such associations in different clinical settings using different types of PPIs and H_2_RA. Finally, we could not investigate the degrees and patterns of protopathic bias related to both PPI and H_2_RA use.

## Conclusions

Initiation of a PPI is associated with a short-term increased risk of MI. However, a similar increased risk is also seen with H_2_RAs. Application of a novel SCCS active comparator method suggests there is no evidence to indicate a higher risk of MI caused by PPIs. Given that this study is an observational study, a body of evidence is warranted before guiding prescribing decisions. More studies are required to explore the feasibility of using active comparators in SCCS to address protopathic bias.

## Ethics approval

The study was approved by the Institutional Review Board of the University of Hong Kong/Hospital Authority Hong Kong West Cluster (IRB reference number: UW 21–197).

## Supplementary Material

dyac196_Supplementary_Data

## Data Availability

The data underlying this article are available in the article and in its online Supplementary material.
