# Correction to: Proton pump inhibitors and myocardial infarction: an application of active comparators in a self-controlled case series

**DOI:** 10.1093/ije/dyac222

**Published:** 2022-11-16

This is a correction to: Celine S L Chui, Ka Shing Cheung, Jeremy P Brown, Ian J Douglas, Ian C K Wong, Esther W Chan, Angel Y S Wong, Proton pump inhibitors and myocardial infarction: an application of active comparators in a self-controlled case series, International Journal of Epidemiology, 2022, dyac196, https://doi.org/10.1093/ije/dyac196

In the published article, the estimates should have been exponentiated after obtaining the total standard error to calculate the confidence intervals in the simple ratio approach. This affected Table 2 and Supplementary Tables S6, S8 and S10. Only the confidence intervals were affected by this error. The corrections did not change the interpretation and conclusions of the study. The last author apologises for the errors.

The paper has been corrected.

