# Supplementary material for: Proton pump inhibitors and myocardial infarction: an application of active comparators in a self-controlled case series
Source: Int J Epidemiol. 2022 Oct 19;52(3):899–907. doi: 10.1093/ije/dyac196 (PMC10244046; doi:10.1093/ije/dyac196)
Supplement: dyac196_Supplementary_Data [file dyac196_supplementary_data.docx]

**Supplementary material: Proton pump inhibitors and myocardial infarction: an application of active comparators in a self-controlled case series**

Contents

[**Method S1. Estimation of incidence rate ratio and 95% confidence interval using simple ratio approach** 3](#_Toc114573880)

[**Method S2. Model in effect modifier approach in Stata.** 4](#_Toc114573881)

[**Table S1. The number of each type of proton pump inhibitor prescription identified in outpatient setting during 2003-2014.** 5](#_Toc114573882)

[**Figure S1: Illustration for self-controlled case series design** 6](#_Toc114573883)

[**Figure S2: Flow of subject inclusion** 7](#_Toc114573884)

[**Table S2. Demographic and health characteristics of the patients on the day of their first outpatient oral proton pump inhibitor and H_2_ receptor antagonist prescription respectively** 8](#_Toc114573885)

[**Table S3. Patterns of days of supply for prescriptions, observation period, risk periods and baseline periods in proton pump inhibitor and H_2_ receptor antagonist users** 9](#_Toc114573886)

[**Table S4. The number of each type of H_2_ receptor antagonist identified in outpatient setting during 2003-2014.** 10](#_Toc114573887)

[**Table S5. Incidence rate ratios for myocardial infarction associated with proton pump inhibitors and H_2_ receptor antagonist respectively in a sensitivity analysis with 2-year band age adjustment** 11](#_Toc114573888)

[**Table S6. Novel self-controlled case series using active comparators in a sensitivity analysis with 2-year band age adjustment** 12](#_Toc114573889)

[**Table S7. Incidence rate ratios for myocardial infarction associated with proton pump inhibitors and H_2_ receptor antagonist respectively in a sensitivity analysis removing overlapped cohort of proton pump inhibitors and H_2_ receptor antagonist.** 13](#_Toc114573890)

[**Table S8. Novel self-controlled case series using active comparators in a sensitivity analysis removing overlapped cohort of proton pump inhibitors and H_2_ receptor antagonist.** 14](#_Toc114573891)

[**Table S9. Incidence rate ratios for myocardial infarction associated with proton pump inhibitors and H_2_ receptor antagonist respectively in a sensitivity analysis removing people who ever prescribed PPI/H_2_RAs respectively 60 days before or after the drug of interest.** 15](#_Toc114573892)

[**Table S10. Novel self-controlled case series using active comparators in a sensitivity analysis removing people who ever prescribed PPI/H_2_RAs respectively 60 days before or after the drug of interest.** 16](#_Toc114573893)

[**Table S11. Incidence rate ratios for myocardial infarction associated with proton pump inhibitors and H_2_ receptor antagonist respectively in a sensitivity analysis removing people who ever prescribed PPI/H_2_RAs respectively in any risk periods.** 17](#_Toc114573894)

[**Table S12. Novel self-controlled case series using active comparators in a sensitivity analysis removing people who ever prescribed PPI/H_2_RAs respectively in any risk periods.** 18](#_Toc114573895)

# **Method S1. Estimation of incidence rate ratio and 95% confidence interval using simple ratio approach**

We calculated the comparator adjusted incidence ratio ratios (IRRs) by dividing (IRRs for PPIs) by (IRRs for H_2_RA) for each risk period. For 95% CI, we first obtained the standard errors of the estimates for each risk period for PPI and H_2_RA analyses respectively. We then estimated the total standard error for each risk window by taking a square root of (addition of the square of standard error of the estimate for PPI and the square of standard error of the estimate for H_2_RA).

We then calculated the 95% confidence interval as follows: IRR ± 1.96* total SE

# **Method S2. Model in effect modifier approach in Stata.**

xtpoisson nevents i.agegroup i.drugtype i.exposed i.drugtype#i.exposed, fe i(patid) offset(loginterval) irr

where nevents is the outcome, agegroup represents age group, drugtype indicates PPI or H2RA, exposed represents the risk window, patid represents an individual, loginterval is the log interval length of each period

# **Table S1. The number of each type of proton pump inhibitor prescription identified in outpatient setting during 2003-2014.**

| **Drug name** | **Number of prescriptions** |
| --- | --- |
| Dexlansoprazole | 2705 |
| Esomeprazole | 382 959 |
| Lansoprazole | 191 468 |
| Omeprazole | 17 140 |
| Pantoprazole | 1 298 028 |
| Rabeprazole | 449 549 |
| Total | 2 341 849 |

# **Figure S1: Illustration for self-controlled case series design**


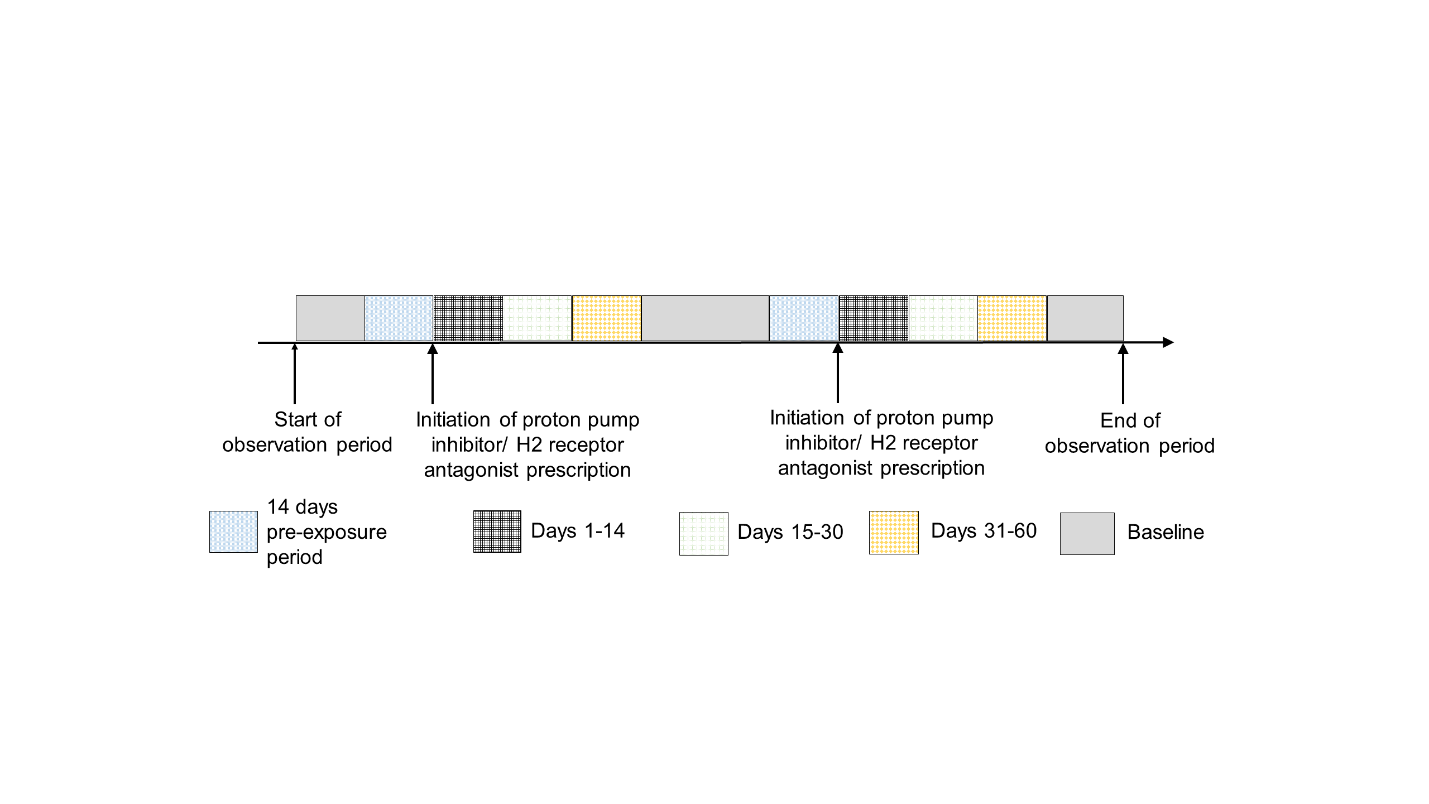


# **Figure S2: Flow of subject inclusion**

**
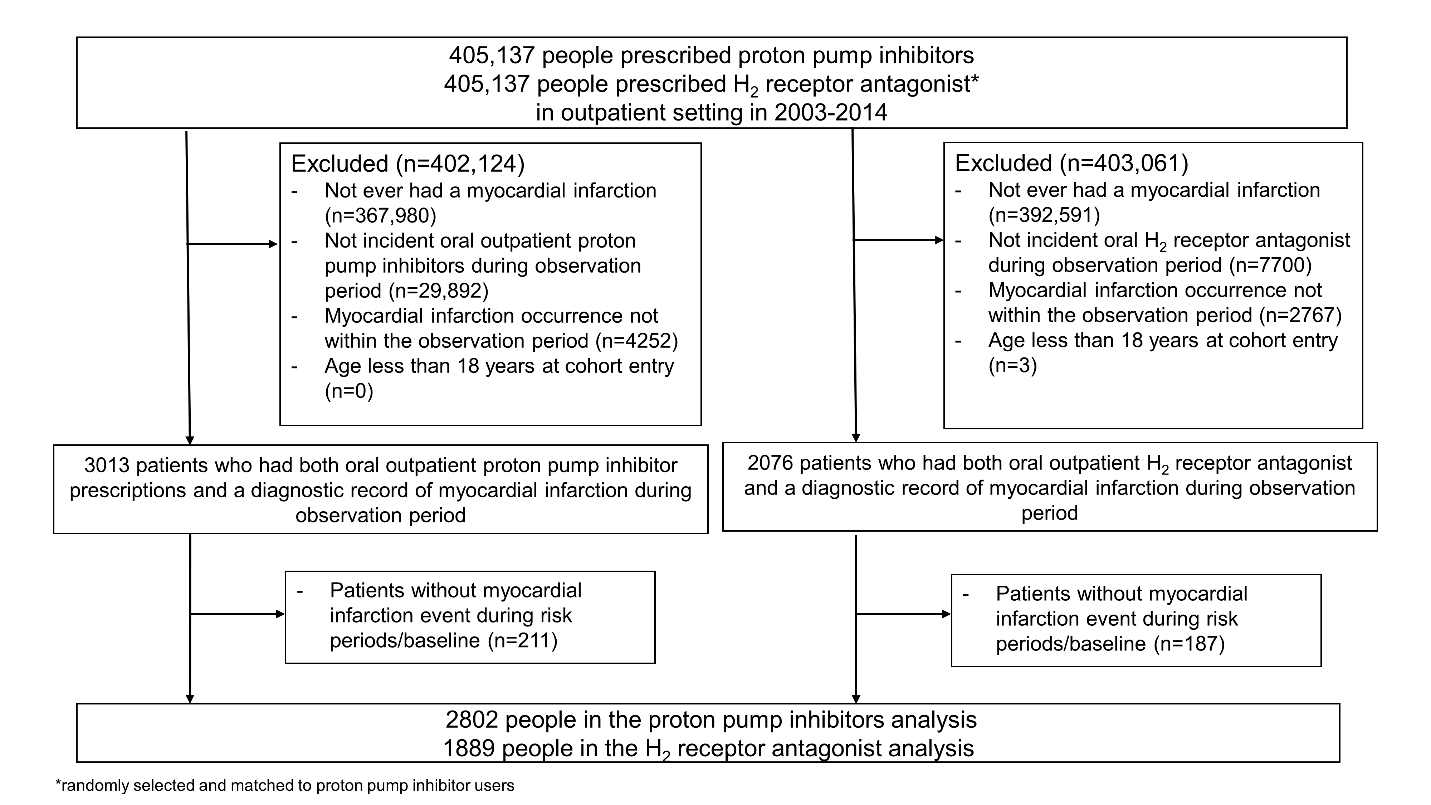
**

# **Table S2. Demographic and health characteristics of the patients on the day of their first outpatient oral proton pump inhibitor and H_2_ receptor antagonist prescription respectively**

|  | **Proton pump inhibitor** | **H_2_ receptor antagonist** |
| --- | --- | --- |
| N | 2802 | 1889 |
| Age, mean (SD) | 68.7 (13.4) | 67.8 (13.2) |
| Men | 64% | 69% |
| *Baseline characteristics N, (%)* | | |
| Chronic obstructive pulmonary disease | 173 (6.2%) | 82 (4.3%) |
| Diabetes | 710 (25.3%) | 357 (18.9%) |
| Hypertensive diseases | 1098 (39.2%) | 568 (30.1%) |
| Ischaemic stroke | 220 (7.9%) | 103 (5.5%) |
| Coronary heart disease  (excluding MI) | 1274 (45.5%) | 623 (33.0) |
| Heart failure | 428 (15.3%) | 223 (11.8%) |
| Venous thromboembolism | 21 (0.8%) | 13 (0.7%) |
| End stage renal failure | 24 (0.9%) | 7 (0.4%) |
| *Drug prescription issued in the past 180 days* |  |  |
| Beta-blockers | 1607 (57.4%) | 965 (51.1%) |
| Calcium channel blockers | 1023 (36.5%) | 618 (32.7%) |
| Nitrates | 1645 (58.7%) | 847 (44.8%) |
| Other anti-angina medications | <5 | <5 |
| Aspirin | 2035 (72.6%) | 1077 (57.0%) |
| Statin | 1627 (58.1%) | 904 (47.9%) |

# **Table S3. Patterns of days of supply for prescriptions, observation period, risk periods and baseline periods in proton pump inhibitor and H_2_ receptor antagonist users**

|  | **PPIs** | **H_2_RAs** |
| --- | --- | --- |
| *Observation period* |  |  |
| Mean | 3292.9 | 3264.3 |
| Median | 3565 | 3531.5 |
| Min, Max | 60, 4383 | 114, 4383 |
| *Baseline period* |  |  |
| Mean | 3325.6 | 3145.5 |
| Median | 3596 | 3395 |
| Min, Max | 31, 4368 | 40, 4368 |
| *14 days pre-exposure period* |  |  |
| Mean | 13.9 | 13.9 |
| Median | 14 | 14 |
| Min, Max | 1, 14 | 1, 14 |
| *Risk period: Days 1-14* |  |  |
| Mean | 13.5 | 13.8 |
| Median | 14 | 14 |
| Min, Max | 1, 14 | 1, 14 |
| *Risk period: Days 15-30* |  |  |
| Mean | 15.6 | 15.7 |
| Median | 16 | 16 |
| Min, Max | 1, 16 | 1, 16 |
| *Risk period: Days 31-60* |  |  |
| Mean | 28.4 | 28.2 |
| Median | 30 | 30 |
| Min, Max | 1, 30 | 1, 30 |
| *Days supplied for prescriptions* |  |  |
| Mean | 207 | 166 |
| Median | 56 | 27 |
| Min, Max | 1, 3300 | 1, 3758 |

# **Table S4. The number of each type of H_2_ receptor antagonist identified in outpatient setting during 2003-2014.**

| **Drug name** | **Number of prescriptions** |
| --- | --- |
| Cimetidine | 9094 |
| Famotidine | 13 137 709 |
| Nizatidine | 12 |
| Ranitidine | 50 252 |
| Total | 13 197 067 |

# **Table S5. Incidence rate ratios for myocardial infarction associated with proton pump inhibitors and H_2_ receptor antagonist respectively in a sensitivity analysis with 2-year band age adjustment**

| **Proton pump inhibitors (N=2802)** | |  |  |
| --- | --- | --- | --- |
|  | **No of events** | **Total person-year** | **Incidence rate ratio (95% CI)** |
| Baseline | 2631 | 23,829 | Ref |
| 14 days  pre-exposure period | 27 | 127 | 0.96 (0.65–1.40) |
| Days 1-14 | 56 | 123 | 2.09 (1.60–2.73) |
| Days 15-30 | 33 | 133 | 1.17 (0.83–1.66) |
| Days 31-60 | 55 | 234 | 1.15 (0.88–1.51) |
| **H_2_ receptor antagonist (N=1889)** | | | |
| Baseline | 1715 | 14,950 | Ref |
| 14 days  pre-exposure period | 6 | 140 | 0.19 (0.09–0.43) |
| Days 1-14 | 67 | 139 | 2.17 (1.69–2.79) |
| Days 15-30 | 31 | 155 | 0.91 (0.64–1.30) |
| Days 31-60 | 70 | 274 | 1.16 (0.91–1.49) |

# **Table S6. Novel self-controlled case series using active comparators in a sensitivity analysis with 2-year band age adjustment**

|  | **Simple Ratio Estimate** | | **Effect Modifier Estimate** | |
| --- | --- | --- | --- | --- |
|  | Estimate | 95%CI | Estimate | 95%CI |
| 14 days  pre-exposure period | 5.05 | 2.08–12.30 | 4.18 | 1.72–10.16 |
| Days 1-14 | 0.96 | 0.67–1.39 | 0.80 | 0.56–1.16 |
| Days 15-30 | 1.29 | 0.78–2.12 | 1.07 | 0.65–1.76 |
| Days 31-60 | 0.99 | 0.69–1.43 | 0.82 | 0.57–1.17 |

# **Table S7. Incidence rate ratios for myocardial infarction associated with proton pump inhibitors and H_2_ receptor antagonist respectively in a sensitivity analysis removing overlapped cohort of proton pump inhibitors and H_2_ receptor antagonist.**

| **Proton pump inhibitors (N=2646)** | |  |  |
| --- | --- | --- | --- |
|  | **No of events** | **Total person-year** | **Incidence rate ratio (95% CI)** |
| Baseline | 2479 | 22,441 | Ref |
| 14 days  pre-exposure period | 27 | 120 | 1.11 (0.75–1.62) |
| Days 1-14 | 54 | 116 | 2.33 (1.77–3.06) |
| Days 15-30 | 32 | 125 | 1.31 (0.92–1.86) |
| Days 31-60 | 54 | 219 | 1.30 (0.98–1.70) |
| **H_2_ receptor antagonist (N=1733)** | | | |
| Baseline | 1571 | 13,767 | Ref |
| 14 days  pre-exposure period | 6 | 127 | 0.24 (0.11–0.53) |
| Days 1-14 | 63 | 126 | 2.53 (1.96–3.28) |
| Days 15-30 | 29 | 140 | 1.06 (0.73–1.54) |
| Days 31-60 | 64 | 247 | 1.34 (1.03–1.73) |

# **Table S8. Novel self-controlled case series using active comparators in a sensitivity analysis removing overlapped cohort of proton pump inhibitors and H2 receptor antagonist.**

|  | **Simple Ratio Estimate** | | **Effect Modifier Estimate** | |
| --- | --- | --- | --- | --- |
|  | Estimate | 95%CI | Estimate | 95%CI |
| 14 days  pre-exposure period | 4.63 | 1.90–11.26 | 4.12 | 1.69–10.03 |
| Days 1-14 | 0.92 | 0.63–1.34 | 0.82 | 0.56–1.19 |
| Days 15-30 | 1.24 | 0.74–2.06 | 1.09 | 0.65–1.81 |
| Days 31-60 | 0.97 | 0.67–1.41 | 0.85 | 0.59–1.24 |

# **Table S9. Incidence rate ratios for myocardial infarction associated with proton pump inhibitors and H_2_ receptor antagonist respectively in a sensitivity analysis removing people who ever prescribed PPI/H_2_RAs respectively 60 days before or after the drug of interest.**

| **Proton pump inhibitors (N=1,566)** | |  |  |
| --- | --- | --- | --- |
|  | **No of events** | **Total person-year** | **Incidence rate ratio (95% CI)** |
| Baseline | 1471 | 13,190 | Ref |
| 14 days  pre-exposure period | 20 | 67 | 1.43 (0.92–2.24) |
| Days 1-14 | 30 | 64 | 2.27 (1.57–3.28) |
| Days 15-30 | 15 | 69 | 1.09 (0.65–1.82) |
| Days 31-60 | 30 | 120 | 1.29 (0.89–1.86) |
| **H_2_ receptor antagonist (N=1663)** | | | |
| Baseline | 1518 | 13,113 | Ref |
| 14 days  pre-exposure period | 5 | 120 | 0.21 (0.09–0.50) |
| Days 1-14 | 52 | 119 | 2.17 (1.64–2.88) |
| Days 15-30 | 25 | 133 | 0.95 (0.63–1.41) |
| Days 31-60 | 63 | 235 | 1.36 (1.05–1.76) |

**Table S10. Novel self-controlled case series using active comparators in a sensitivity analysis removing people who ever prescribed PPI/H2RAs respectively 60 days before or after the drug of interest.**

|  | **Simple Ratio Estimate** | | **Effect Modifier Estimate** | |
| --- | --- | --- | --- | --- |
|  | Estimate | 95%CI | Estimate | 95%CI |
| 14 days  pre-exposure period | 6.81 | 2.54–18.26 | 5.78 | 2.15–15.46 |
| Days 1-14 | 1.05 | 0.66–1.66 | 0.87 | 0.55–1.38 |
| Days 15-30 | 1.15 | 0.60–2.20 | 0.95 | 0.50–1.82 |
| Days 31-60 | 0.95 | 0.60–1.49 | 0.78 | 0.50–1.22 |

# **Table S11. Incidence rate ratios for myocardial infarction associated with proton pump inhibitors and H_2_ receptor antagonist respectively in a sensitivity analysis removing people who ever prescribed PPI/H_2_RAs respectively in any risk periods.**

| **Proton pump inhibitors (N=1,953)** | |  |  |
| --- | --- | --- | --- |
|  | **No of events** | **Total person-year** | **Incidence rate ratio (95% CI)** |
| Baseline | 1831 | 16,451 | Ref |
| 14 days  pre-exposure period | 23 | 84 | 1.29 (0.85–1.95) |
| Days 1-14 | 40 | 81 | 2.38 (1.73–3.27) |
| Days 15-30 | 24 | 86 | 1.37 (0.91–2.06) |
| Days 31-60 | 35 | 149 | 1.19 (0.85–1.67) |
| **H_2_ receptor antagonist (N=1,743)** | | | |
| Baseline | 1586 | 13,659 | Ref |
| 14 days  pre-exposure period | 5 | 125 | 0.20 (0.08–0.47) |
| Days 1-14 | 60 | 124 | 2.39 (1.83–3.11) |
| Days 15-30 | 27 | 139 | 0.98 (0.67–1.44) |
| Days 31-60 | 65 | 245 | 1.34 (1.04–1.73) |

# **Table S12. Novel self-controlled case series using active comparators in a sensitivity analysis removing people who ever prescribed PPI/H_2_RAs respectively in any risk periods.**

|  | **Simple Ratio Estimate** | | **Effect Modifier Estimate** | |
| --- | --- | --- | --- | --- |
|  | Estimate | 95%CI | Estimate | 95%CI |
| 14 days  pre-exposure period | 6.45 | 5.48–7.42 | 5.61 | 2.12–14.84 |
| Days 1-14 | 1.00 | 0.58–1.41 | 0.85 | 0.56–1.28 |
| Days 15-30 | 1.40 | 0.84–1.96 | 1.20 | 0.68–2.09 |
| Days 31-60 | 0.89 | 0.46–1.31 | 0.75 | 0.49–1.15 |
